# Have orthodontists changed their retention and follow-up protocols due to contemporary orthodontics? An online survey

**DOI:** 10.34172/joddd.41030

**Published:** 2024-09-07

**Authors:** Liliana Ávila Maltagliati, Sandra Maria Mesquita Alves Uchôa, Rogéria Cristina Calastro, Mayara Paim Patel, Ana Carla Raphaelli Nahás, Hélio Doyle Pereira da Silva, Murilo Matias

**Affiliations:** ^1^Department of Orthodontics, Faculty of Dentistry, Guarulhos University, São Paulo, Brazil; ^2^Department of TMJ disorder, Faculty of Dentistry, Uninassau University, Pernambuco, Brazil; ^3^Private Office, Brasília, Brazil

**Keywords:** Age factors, Clinical protocols, Corrective orthodontics, Orthodontic retainers

## Abstract

**Background.:**

With advancements in orthodontic technology, treatment durations have shortened, often concluding at earlier ages. This shift prompts scrutiny of contemporary retention and post-treatment protocols. The study aimed to assess current professional preferences, compare them against patient age and treatment duration, and investigate the potential impacts of reduced treatment times on professional protocols, particularly when treatment concludes before pubertal growth.

**Methods.:**

A questionnaire comprising 12 multiple-choice questions focused on active treatment and retention phases was developed using an online survey platform. It was distributed to licensed orthodontists engaged in patient treatment. Bivariate analysis was conducted using ANOVA and the Kruskal-Wallis test, with pairwise comparisons facilitated by the Dwass-Steel-Critchlow-Fligner method.

**Results.:**

Of 743 respondents, representing a 32% response rate, approximately 70% reported initiating treatment with fixed appliances in pre-pubertal patients. The most prevalent treatment combination involved commencing treatment during early permanent dentition and lasting between 12 to 24 months, resulting in treatment completion before full growth maturation. No discernible individualization was observed in retention protocols or post-retention follow-ups. Traditional retainer prescription post-orthodontic therapy was unanimous among respondents. Notably, experienced orthodontists tended to prefer regular patient visits for follow-up, while less experienced counterparts discharged patients after 12 months.

**Conclusion.:**

Contemporary orthodontic treatments are characterized by shorter durations, yet orthodontists have not adopted retention and post-treatment follow-up practices accordingly. There is a pressing need for evidence-based guidelines to develop protocols tailored to the shorter treatment durations and the increasing prevalence of younger patients completing treatment.

## Introduction

 Comprehensive orthodontic treatment has traditionally been perceived as lengthy, shaped by the biological principles underlying optimal tooth movement. With the advent of cutting-edge technologies in orthodontic materials and the integration of skeletal anchorage, treatment durations have notably reduced, often concluding at earlier stages of life. There is an unquestionable tendency among orthodontists to reduce the duration of orthodontic treatment, which holds potential benefits for practitioners and patients. Prolonged treatment durations have historically been associated with heightened risks of iatrogenic complications stemming from oral appliance therapy, including root resorption, plaque-induced conditions, and demineralization.^1^

 The short treatment duration experienced by younger patients necessitates the early implementation of retention appliances, often preceding the completion of craniofacial growth. Retention devices play a crucial role in mitigating post-treatment instability and relapse, and types of appliances and use recommendations vary among professionals.^2,3^

 Undoubtedly, the retention phase holds equal significance to the treatment in preserving the outcomes of malocclusion correction and ensuring enduring stability. Orthodontic treatment stability is predominantly influenced by three primary factors: the time required to reorganize gingival and periodontal tissues, the inherent instability of teeth following orthodontic intervention, and the dynamic changes induced by growth.^4^ Dental arches undergo continual transformations from the deciduous dentition phase through adulthood, characterized by individual variations attributable to the biological migration of dentition. These changes often manifest as anterior crowding, particularly pronounced in the mandible.^5^

 Traditionally, fixed lingual retainers bonded to the mandibular anterior teeth have been a prevalent choice for several years post-orthodontic treatment, or at a minimum, until growth ceases. Lifelong retention has been increasingly prescribed instead of retention for a limited time.^6^ While there exists a prevailing consensus that prolonged retention is optimal for preserving treatment outcomes, empirical evidence to substantiate recommendations on orthodontic retention protocols remains somewhat lacking, particularly given contemporary short treatment durations, the age at treatment initiation and conclusion, as well as variations in malocclusion types and gender considerations.^7^

 As advancements continue to facilitate shorter treatment times, concluding corrective interventions while patients are still undergoing growth, concerns regarding stability and growth-induced changes have come to the forefront. This study thus seeks to ascertain whether orthodontists have adopted the types and duration of retention appliance usage and post-treatment follow-up protocols to formulate more personalized approaches for these evolving patient demographics.

## Methods

 This study underwent ethical review and received approval from the Research Ethics Committee under reference number 4.202.572/2020, adhering to the principles outlined in the Helsinki Declaration. A comprehensive survey instrument, comprising 12 multiple-choice questions categorized into three distinct sections representing pertinent information, was developed using an online survey platform (i.e., Google Forms application). The questionnaire was disseminated via email and popular instant messaging applications such as WhatsApp and Facebook to licensed orthodontists actively engaged in patient treatment.

 A sample calculation was conducted with a minimum test power of 80% and a confidence level of 95% to determine the minimum required number of responses. Accounting for a population of 30,000 orthodontic specialists, it was established that a minimum of 380 responses would be necessary to achieve the desired statistical significance.

 To ensure comprehensive participation, reminders were dispatched at regular intervals, with messages and emails resent up to three times within a 15-day timeframe to prompt responses from those yet to engage with the survey.

 Before engaging with the questionnaire, all the participants had to accept the invitation to participate after reading the consent terms. The questionnaire was structured into three distinct sections, delineated as follows:

###  Section 1: Sociodemographic status and practice experience of respondents

 The respondents were asked to furnish details regarding their gender, workplace affiliation, and the duration of their orthodontic practice post-completion of postgraduate studies.

###  Section 2: Initial age of treatment and duration of treatment 

 The respondents were asked about the patient’s age or period of occlusion. This section of the questionnaire focused on eliciting responses pertaining to the commencement age or developmental stage of occlusion when corrective treatment was initiated, as well as the average duration of such treatment.

###  Section 3: Retention protocol

 This section of the study examined the types of retainers typically used by orthodontists, their post-treatment monitoring protocols, and any personalized approaches related to age and treatment duration.

 Univariate descriptive statistical analysis was employed to analyze qualitative variables, with categorical data expressed as percentages across respective categories. ANOVA and the Kruskal-Wallis test were used for bivariate analysis, followed by the Dwass-Steel-Critchlow-Fligner test for pairwise comparisons. All the statistical analyses were conducted using Jamovi software (The Jamovi Project 2021, Version 1.6), with significance set at P < 0.05.

## Results

 Of 2000 emails and 300 instant messages dispatched, the questionnaires garnered 743 responses, representing just over 32% of the outreach. Despite surpassing the required number for statistical tests, every response was retained, given the qualitative nature of the experiment and the questionnaire’s multiple-choice format.

Table 1 provides an overview of the sample’s demographic breakdown, delineating gender distribution and clinical experience.

**Table 1:** Sample size, gender, and professional experience

**Variable**	**No.**	**%**
Gender		
Male	208	27.96
Female	535	72.04
Experience in orthodontic practice		
< 10 years	272	36.56
10‒20 years	311	41.94
≥ 20 years	160	21.50

Table 2 elucidates the relationship between treatment duration and the initial age of treatment initiation with fixed appliances. Notably, while it was anticipated that treatments commencing at earlier stages might necessitate longer durations to accommodate growth and the completion of permanent dentition, the majority of respondents across all treatment initiation periods cited treatment durations spanning two to three years.

**Table 2:** Distribution of treatment beginning period according to treatment duration (months)

		**How long does fixed orthodontic treatment last, on average?**				
		**<12 mon**	**12‒24 mon**	**24‒30 mon**	**≥30 mon**	**Total**
When fixed orthodontic treatment starts on average?	Mixed dentition	3 (0.40%)	152 (20.46%)	102 (13.3%)	11 (1.48%)	268 (36.07%)
	Early permanent dentition	0 (0.00%)	172 (23.15%)	77 (10.36%)	3 (0.40%)	252 (33.92%)
	Pubertal period	3 (0.40%)	95 (12.79%)	66 (8.88%)	2 (0.27%)	166 (22.34%)
	Post-pubertal period	1 (0.13%)	43 (5.79%)	13 (1.75%)	0 (0.00%)	57 (7.67%)
Total		7 (0.94%)	462 (62.18%)	258 (34.72%)	16 (2.15%)	743 (100%)

Tables 3 and 4 delineate the distribution of the most frequently cited preferences for retention or post-treatment control categorized by treatment duration and initial treatment age. No difference was found in retention protocol preference, irrespective of treatment duration or the age at which treatment commenced, highlighting no individualization concerning patients before or after growth ceasing and fast or prolonged treatment. The only significant difference found was pertaining to the duration of retention and the frequency of follow-up appointments based on the period of treatment commencement. Table 5 further elucidates the predominant preferences in accordance with professional experience. Intriguingly, it appears that orthodontists’ experience in the field does not substantially alter their retention protocol preferences.

**Table 3:** Comparison between protocols for retention (most prevalent answers) and follow-up according to treatment duration (in months)

		**How long does fixed orthodontic treatment last, on average?**					***P*** ** value (Kruskal-Wallis test)**
		**<12 mon**	**12‒24 mon**	**24‒30 mon**	**≥30 mon**	**Total**	
Retention protocol	Upper removable appliance and lower fixed 3x3 retainer	5 (0.69%)	376 (51.72%)	215 (29.57%)	11 (1.51%)	607 (83.49%)	0.086
Removable retainer most frequently used	Hawley retainer in100% of the cases	3 (0.41%)	163 (22.42%)	128 (17.61%)	5 (0.69%)	299 (41.13%)	0.459
How long upper removable retainer is advised to stay in place	For 12 months	2 (0.27%)	197 (27.98%)	115 (15.82%)	5 (0.69%)	328 (44.15%)	0.815
	For 5 years	2 (0.27%)			5 (0.69%)		
	Permanently	2 (0.27%)					
Fixed 3 × 3 retainer, which are most frequently used	Rigid, bonded to canines	2 (0.27%)				236 (31.77%)	0.116
	Rigid, bonded to all teeth	2 (0.27%)					
	Rigid, hygienic	2 (0.27%)	139 (19.12%)	85 (11.69%)	6 (0.82%)		
How long lower fixed retainer is advised to stay in place	Permanently	6 (0.82%)	360 (49.52%)	213 (29.30%)	10 (1.37%)	589 (81.02%)	0.354
Post-treatment follow-up protocol	Follow-up visits for 12 months, then discharge of the patient	3 (0.41%)	160 (22.00%)		8 (1.10%)	258 (34.73%)	0.655
	Follow-up visits for 5 years, then discharge of the patient			87 (11.97%)			

**Table 4:** Comparison between protocols for retention and follow-up (most prevalent answers) according to initial treatment age

		**When does fixed orthodontic treatment start on average?**					***P*** ** value (Kruskal-Wallis test)**
		**Mixed dentition**	**Early permanent dentition**	**Pubertal period**	**Post-pubertal period**	**Total**	
Retention protocol	Upper removable appliance and lower fixed 3x3 retainer	206 (28%)	215 (29.57%)	140 (19.25%)	46 (6.33%)	607 (83.49%)	0.644
Removable retainer most frequently used	Hawley retainer in 100% of the cases		115 (15.82%)	71 (9.77%)	20 (2.75%)	305 (41.06%)	0.373
	Hawley retainer in more than 50% and thermoplastic in less than 50%	99 (13.62%)					
How long upper removable retainers are advised to stay in place	For 12 months	104 (14.30%)	121 (16.64%)	69 (9.49%)	25 (3.44%)	319 (43.88%)	0.171
Fixed 3x3 retainer most frequently used	Rigid, bonded to all anterior teeth	75 (10.32%)				234 (31.50%)	0.412
	Rigid, hygienic		77 (10.59%)	57 (7.84%)	25 (3.44%)		
How long lower fixed retainers are advised to stay in place	Permanently	192 (26.41%) ^a^	209 (28.75%) ^ab^	142 (19.53%) ^b^	46 (6.33%) ^ab^	589 (81.02%)	0.015*
Post-treatment follow-up protocol	Follow-up visits for 12 months, then discharge of the patient	109 (14.99%) ^a^			21 (2.89%) ^ac^	304 (40.92%)	< 0.001*
	Follow-up visits for 5 years, then discharge of the patient		91 (12.52%) ^ab^	62 (8.53%) ^b^			
	Follow-up visits permanently				21 (2.89%) ^ac^		

*Statistically significant. Letters a, b, c: Dwass-Steel-Critchlow-Fligner pairwise comparisons. Statistically significant for different letters.

**Table 5:** Comparison between protocols for retention (most prevalent) and follow-up according to professional experience

		**Professional experience**				***P*** ** value (Kruskal-Wallis test)**
		**<10 y**	**≥10 y and<20 y**	**≥20 y**	**Total**	
Period when fixed orthodontic treatment start	Mixed Dentition	143 (19.35%)	135 (18.23%)		342 (46.05%)	0.503
	Early permanent dentition			64 (8.60%)		
Treatment duration	24–30 m	136 (18.28%)	143 (19.36%)	112 (15.09%)	391 (52.63%)	0.623
Retention protocol	Upper removable appliance and lower fixed 3 × 3 retainer	247 (33.35%)	274 (36.92%)	125 (16.88%)	646 (86.95%)	0.740
Removable retainer most frequently used	Hawley retainer in 100% of the cases	136 (18.31%)	152 (20.19%)	80 (10.62%)	368 (49.53%)	0.825
How long upper removable retainers are advised to stay in place	For 12 months	119 (16.01%)	112 (15.07%)		407 (54.77%)	0.209
	For 5 years		112 (15.07%)	64 (8.61%)		
Fixed 3x3 retainer most frequently used	Rigid, bonded to canines			56 (7.53%)	437 (58.81%)	0.605
	Rigid, bonded to all anterior teeth	103 (13.86%)				
	Rigid, hygienic	103 (13.86%)	119 (16.01%)	56 (7.53%)		
How long lower fixed retainers are advised to stay in place	Permanently	223 (30.01%)	239 (32.16%)	127 (17.09%)	589 (81.02%)	0.998
Post-treatment follow-up protocol	Follow-up visits for 12 months, then discharge of the patient	88 (11.84%)			288 (38.76%)	0.141
	Follow-up visits for 5 years, then discharge of the patient		128 (17.22%)	72 (9.69%)		

## Discussion

 The primary objective of this paper was to investigate whether orthodontists have implemented any revisions in retention and post-treatment follow-up protocols in recent times and to ascertain any correlation between the average age of patients at treatment onset and the current treatment duration. Despite advancements in specialty mechanics and material evolution aimed at shortening treatment durations, our findings, consistent with existing literature, suggest the persistence of traditional practices in daily orthodontic routines.^6,8^

 While not the primary focus of this study, an ancillary inquiry explored whether clinical experience and gender influenced orthodontists’ clinical preferences. Notably, the sample distribution (Table 1) revealed a predominant representation of females (72.04% female and 27.96% male), precluding a gender-based comparison. However, the distribution of years of experience was more balanced, enabling meaningful comparisons (Table 5).

 The intersection of treatment durations spanning from 12 to 24 months and commencing treatment during the early permanent dentition phase accounted for over 50% of cases, with an additional 20.46% commencing treatment even earlier, while some deciduous teeth were still present (Table 2). This finding mirrors similar observations reported in other studies, underlining the prevalence of initiating orthodontic treatment at earlier developmental stages. Consequently, a significant proportion of patients completed corrective treatment during puberty or even before the onset of the pubertal growth spurt before achieving full skeletal maturity. Despite the substantive implications of this trend, the retention and follow-up protocol preferences among orthodontists remained broadly consistent, indicating a prevailing uniformity of approach among respondents (Tables 2 and 3).

 The most favored combination among orthodontists was a removable retainer in the upper arch and a fixed 3 × 3 one in the lower arch (83.49%). Nearly half of the specialists reported employing the Hawley appliance in 100% of their cases (299 out of 743 orthodontists). The remaining half used both Hawley and vacuum retainers, with a higher prevalence among those employing the Hawley appliance in over 50% of cases (31.91%). Despite the burgeoning popularity of invisible retainers in recent years, evidenced by their increasing adoption rates until 2011,^9,10^ it is noteworthy that Hawley retainers emerged as the most widely used in the United States, with a usage rate of 47%, closely paralleling our current findings (41.13%). According to Pratt et al,^11^ this phenomenon may confer certain advantages, as patients wearing Hawley appliances tend to exhibit greater compliance with their use over an extended duration. This stands in contrast to orthodontic practices in the United Kingdom, Australia, and New Zealand, where orthodontists tend to prefer vacuum-formed retainers for the upper arch.^9^

 Irrespective of the type of retention employed, a substantial majority (43.99%) of respondents advocate for its usage over 12 months. The remaining preferences were divided between those recommending usage for five years and those advocating it for permanent retention, indicative of entrenched traditional practices and lingering uncertainty regarding the optimal course of action. Notably, the existing literature on protocols governing the duration of removable retainer use remains relatively scant. Indeed, there is a pressing need for more robust evidence to underpin recommendations pertaining to the most efficacious retention protocols.^12^

 The adoption of mandibular fixed retention appears nearly unanimous, with 83% of respondents indicating its use in the lower arch. This protocol was the choice of at least half of the orthodontists in the United States.^9^

 However, selecting the specific fixed retention type remains controversial. Our findings revealed that one-third of clinicians favored the hygienic retainer, although other options were closely trailing behind. Notably, the literature suggests that the hygienic retainer offers commendable stability and minimal long-term changes, further complicating the decision-making process.^13^ The absence of a clear preference among clinicians may be attributed to the shortage of high-quality evidence delineating the superiority of one retainer over others or prescribing a definitive retention regimen. Consequently, clinicians’ decision-making is likely influenced by a combination of their clinical experience, expertise, and patient-specific factors, including expectations and circumstances.^7^

 The recommendation to maintain the fixed 3 × 3 retainer endlessly was quite unanimous (81% of the respondents) (Tables 3, 4, and 5) and aligns seamlessly with findings in the existing literature.^10,14^ It means that, in most cases, patients will grow with a retainer in place, raising pertinent questions regarding the necessity for individualized approaches based on factors such as malocclusion type, patient gender, and age at treatment conclusion.

 From the standpoint of safeguarding treatment outcomes, stability, and oral health, it is noteworthy that while many orthodontists endorse lifelong retention, particularly in the lower arch, the majority do not adhere to regular patient follow-ups beyond five years.^9,11^ Although there appears to be a trend toward extending post-treatment follow-up periods relative to professional experience, this trend failed to attain statistical significance (Table 5).

 Modern technical procedures have significantly reduced the treatment duration.^15^ However, despite these innovations, orthodontists remain divided regarding the necessity for retention, the choice of retainer type, and the optimal duration of retainer wear. Particularly noteworthy is the lack of differentiation in retention protocols between adolescent and adult patients despite the complexities posed by concluding treatment in individuals still undergoing growth.^2,3,16-18^ In a recent retrospective investigation, class II patients who used lower fixed retainers for an average duration of 2.7 years were compared to those who received no retention and to untreated patients. Notably, during a long-term evaluation conducted 6 and 12 years post-treatment, no discernible differences in incisor irregularity were observed between the groups, with the authors recommending life-long retention for the mandibular anterior segment.^19^

 Many practitioners recommend life-long retention, but relevant evidence is missing on the potential side effects after such a long retention time. Certain post-treatment side effects have been documented, such as buccal root torque, which can result in bone fenestration and pose a risk to the periodontal health of affected teeth. While authors hypothesize that these occurrences may stem from inadequately adapted retention strategies, conclusive evidence supporting this assertion remains elusive.^14,19,20^ Probably, the poor quality of studies on mandibular alignment changes after orthodontic treatment and the lack of prospective studies contributed to it, in addition to the heterogeneity of factors influencing the alignment of mandibular anterior teeth in the long term.^21^

 It is imperative to acknowledge the potential occurrence of mandibular incisor crowding during adolescence, typically observed between the ages of 13 and 18. This phenomenon, often regarded as a late manifestation of primary crowding, is primarily attributed to maturational factors.^22-24^ Furthermore, late mandibular crowding may manifest after 18 years of age, potentially driven by disproportionate mandibular growth compared to the maxilla, coupled with anterior rotation. A recent study encompassing patients aged between 12 and 21 years underscored a notable mandibular growth trajectory relative to the maxilla. This growth pattern was accompanied by dentoalveolar adaptations, including retroclination of the lower incisors, proclination of the upper incisors in males, and a reduction in lower dental arch length.^25^ Similar findings have been corroborated by previous research, particularly emphasizing gender-specific variations.^26,27^ It is crucial to recognize that occlusion is a dynamic developmental process throughout life, exhibiting significant individual variability. Therefore, when assessing post-retention changes in occlusion, it is imperative to contextualize these findings within the broader framework of natural growth changes.^2,5^

 Notably, the subjects included in these studies predominantly exhibited Cl I or II malocclusions, with no representation of Cl III cases. Furthermore, the participants varied in age at which their orthodontic treatment commenced. Notably, there was no differentiation based on sex or growth pattern among the subjects, underscoring the need for further research encompassing diverse demographic profiles to comprehensively understand the implications of mandibular growth and its impact on post-retention occlusal changes.^9,11,14,19,21^ All these might influence the results because some disorders are more linked to late mandibular growth problems. Therefore, treating all patients in the post-retention phase similarly may lead to poor outcomes, especially for males and those with Cl III skeletal discrepancies, as these are known to present related problems during the post-pubertal phase.^28-30^

 If lifelong retention is deemed the preferred method, it is strongly advised to implement a structured, continuous follow-up schedule to meticulously monitor retainers as an integral component of a patient’s routine dental examinations aimed at identifying and addressing any potential issues.^31^ Additionally, there is a pressing need for research to develop individualized retention protocols, considering factors such as age, gender, type of malocclusion, and growth patterns. These studies should endeavor to identify the most effective retention modalities for specific orthodontic conditions, ultimately providing robust support for establishing clinical practice guidelines governing personalized orthodontic retention protocols and post-retention follow-up procedures.

 As an online survey, certain limitations were inherent, including relying on subjective responses reflecting professionals’ opinions rather than direct clinical practice observations. Additionally, the respondents were tasked with providing estimations of their patients’ average treatment duration and initial age, introducing a degree of approximation that may lead to some data being either overestimated or underestimated. Moreover, the nature of the research precluded a direct comparison between various retention modalities, their duration of use, and their impact on treatment stability, relapse, or potential iatrogenic tooth movements. Therefore, it is strongly recommended that future studies include clinical trials specifically designed to address these objectives, providing more conclusive insights into the efficacy and outcomes of different retention protocols.

## Conclusion

 The retention protocol and post-treatment follow-up procedures observed in this study did not mirror the advancements in orthodontic practices aimed at reducing treatment duration. Notably, factors such as the initial and final age of orthodontic treatment, treatment duration, and practice experience did not exert discernible influence on orthodontists’ preferences for retention appliances or follow-up protocols.

## Acknowledgments

 We extend our sincere gratitude to the Brazilian Association of Orthodontists for their invaluable support in facilitating the distribution of emails to active orthodontic specialists engaged in patient treatment. Their assistance was instrumental in reaching our target audience and ensuring the success of this study.

## Competing Interests

 The authors certify that they have no affiliations with or involvement in any organization or entity with any financial interest (such as honoraria, educational grants, participation in speakers’ bureaus, membership, employment, consultancies, stock ownership, or other equity interest, and expert testimony or patent-licensing arrangements), or non-financial interest (such as personal or professional relationships, affiliations, knowledge or beliefs) in the subject matter or materials discussed in this manuscript.

## Ethical Approval

 This study was approved by Guarulhos University Ethics Committee, number 4.202.572/2020.
